# Weight change and sulfonylurea therapy are related to 3 year change in microvascular function in people with type 2 diabetes

**DOI:** 10.1007/s00125-020-05125-4

**Published:** 2020-03-17

**Authors:** Francesco Casanova, Kim M. Gooding, Angela C. Shore, Damilola D. Adingupu, David Mawson, Claire Ball, Christine Anning, Kunihiko Aizawa, Philip E. Gates, W. David Strain

**Affiliations:** 1 Diabetes and Vascular Medicine Research Centre, Institute of Biomedical and Clinical Science and University of Exeter College of Medicine and Health, Royal Devon & Exeter Hospital, Barrack Road, Exeter, EX2 5AX UK; 2 NIHR Exeter Clinical Research Facility, Royal Devon & Exeter NHS Foundation Trust and University of Exeter, College of Medicine and Health, Exeter, UK

**Keywords:** Diabetes, Epidemiology, Microvascular, Population, Weight loss

## Abstract

**Aims/hypothesis:**

Although cardiovascular disease is the biggest cause of death in people with diabetes, microvascular complications have a significant impact on quality of life and financial burden of the disease. Little is known about the progression of microvascular dysfunction in the early stages of type 2 diabetes before the occurrence of clinically apparent complications. We aimed to explore the determinants of endothelial-dependent and -independent microvascular function progression over a 3 year period, in people with and without both diabetes and few clinical microvascular complications.

**Methods:**

Demographics were collected in 154 participants with type 2 diabetes and in a further 99 participants without type 2 diabetes. Skin microvascular endothelium-dependent response to iontophoresis of acetylcholine and endothelium-independent responses to sodium nitroprusside were measured using laser Doppler fluximetry. All assessments were repeated 3 years later.

**Results:**

People with type 2 diabetes had impaired endothelial-dependent microvascular response compared with those without (AUC 93.9 [95% CI 88.1, 99.4] vs 111.9 [102.3, 121.4] arbitrary units [AU] × min, *p* < 0.001, for those with vs without diabetes, respectively). Similarly, endothelial-independent responses were attenuated in those with diabetes (63.2 [59.2, 67.2] vs 75.1 [67.8, 82.4] AU × min, respectively, *p* = 0.002). Mean microvascular function declined over 3 years in both groups to a similar degree (*p*_interaction_ 0.74 for response to acetylcholine and 0.69 for response to sodium nitroprusside). In those with diabetes, use of sulfonylurea was associated with greater decline (*p* = 0.022 after adjustment for co-prescriptions, change in HbA_1c_ and weight), whereas improving glycaemic control was associated with less decline of endothelial-dependent microvascular function (*p* = 0.03). Otherwise, the determinants of microvascular decline were similar in those with and without diabetes. The principal determinant of change in microvascular function in the whole population was weight change over 3 years, such that those that lost ≥5% weight had very little decline in either endothelial-dependent or -independent function compared with those that were weight stable, whereas those who gained weight had a greater decline in function (change in endothelial-dependent function was 1.2 [95% CI −13.2, 15.7] AU × min in those who lost weight; −15.8 [−10.5, −21.0] AU × min in those with stable weight; and −37.8 [−19.4, −56.2] AU × min in those with weight gain; *p*_trend_ < 0.001). This association of weight change with change in endothelial function was driven by people with diabetes; in people without diabetes, the relationship was nonsignificant.

**Conclusions/interpretation:**

Over 3 years, physiological change in weight was the greatest predictor of change in microvascular function.

**Electronic supplementary material:**

The online version of this article (10.1007/s00125-020-05125-4) contains peer-reviewed but unedited supplementary material, which is available to authorised users.

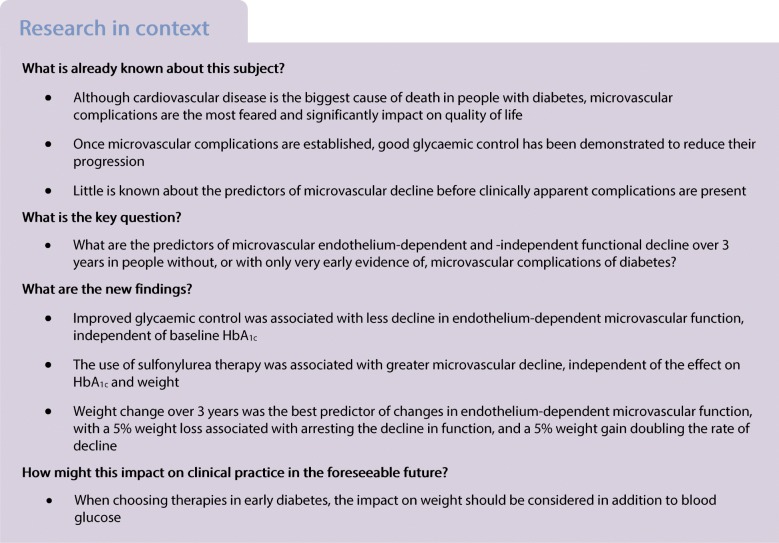



## Introduction

In recent years there has been considerable focus on the impact of type 2 diabetes on cardiovascular disease (CVD) as the principal cause of death in people with diabetes. Microvascular complications of diabetes, however, have a major impact on the quality of life of people with diabetes and are the most feared in people with diabetes [1]. Further, both structural and functional changes in the microvasculature significantly alter the peripheral resistance and thus haemodynamic stress on the heart and other organs. It is thought that this is how they play an independent part in the initiation and progression of atherosclerotic plaques [2].

Whereas the pathophysiological mechanisms of large artery disease have been well studied, the physiology, mechanisms and functional changes over time at the microvascular level are poorly understood. It is known that determinants of microvascular disease progression differ from those at the macrovascular level in type 2 diabetes and thus findings in the large vessels cannot be extrapolated to small vessels [3]. The principal determinants of microvascular disease progression appear to be hyperglycaemia, and resultant advanced glycaemic end-products (AGEs) and hyperinsulinaemia in those with diabetes [4]. Eutrophic (inward) remodelling within the microcirculation in response to hypertension reduces the flow through the vessel, thereby increasing the resistance (itself a driver for further hypertension), and limits potential for endothelially mediated relaxation of the vessels [5]. Conversely, hypertrophic (outward) remodelling maintains the flow, but limits the diffusion capacity of the vessels. The respective causes of these alterations are poorly understood; however, both result in attenuated myogenic responses to sheer stress [6]. The clinically measurable impact is an increased urinary AER. It is important to note that the urinary albumin is not itself a disease progression determinant, but rather a measure of systemic microvascular dysfunction.

Urinary AER, often regarded as the quantifiable hallmark of diabetic microvascular disease, represents the presence of substantial microvascular impairment [7, 8]. The natural history of preclinical changes is poorly understood because of the paucity of long-term studies in relevant surrogates. The techniques used to explore preclinical microvascular dysfunction have developed substantially in recent times [9]. The regulatory mechanisms for the skin microcirculation appear to be different from forearm blood flow [10], and responses in these two vascular territories do not normally correlate in healthy individuals [11, 12]. The use of laser Doppler fluximetry with iontophoresis of acetylcholine (ACh) and sodium nitroprusside (SNP) enables mechanistic studies to non-invasively elucidate endothelial-dependent and -independent effects [13, 14]. Skin microvascular responses have been demonstrated to be reduced in people with type 2 diabetes [7], and associated with left ventricular hypertrophy [15], urinary AER [16], retinopathy [17], coronary artery disease [18] and symptomatic angina independent of atherosclerosis [19]. A recent systematic review and network analysis found progressive microvascular impairment across the spectrum of metabolic health, from a healthy population to those with obesity, glucose impairment and diabetes mellitus [20].

We aimed to explore the determinants of endothelial-dependent and -independent microvascular function progression over a 3 year period, in people both with and without diabetes and few clinical microvascular complications.

## Methods

This study conformed to the Declaration of Helsinki and was approved by the National Research Ethics Service Southwest (10/H0206/67). All participants gave informed written consent.

### Study population

Participants were adult men and women, enhanced with individuals with type 2 diabetes and/or proven CVD recruited as part of the ‘SUrrogate markers for Micro- and Macro-vascular hard endpoints for Innovative diabetes Tools’ (SUMMIT) programme (Innovative Medicines Initiative [IMI] grant number 115006; http://www.imi-summit.eu) via the Peninsula Research Bank, part of the National Institute for Health Research (NIHR) Exeter Clinical Research Facility, and the Royal Devon and Exeter National Health Service (NHS) clinical service.

Type 2 diabetes was diagnosed according to current guidelines by the patients’ physicians based on an HbA_1c_ above 48 mmol/mol (6.5%). Patients diagnosed with type 2 diabetes under the age of 35 or treated with insulin within 12 months of diagnosis were not included in the study. CVD was defined as previously described [7], and this included a medical history of myocardial infarction, percutaneous coronary intervention, coronary arterial bypass graft, unstable angina and specialist-diagnosed cerebrovascular events from stroke and cardiology units and clinics at the Royal Devon and Exeter Hospital, UK. Exclusion criteria included any malignancy requiring active treatment, any treated chronic inflammatory disease, renal replacement therapy and end-stage renal disease.

### Study visits

All participants had multiple visits for both baseline and follow-up assessments 3 years apart (mean follow-up time: 3.14 ± 0.21 years). For both baseline and follow-up, the microvascular studies were organised at the same time of day or as close to this as possible.

All participants refrained from eating and drinking (except water) for at least 2 h before the visit, and avoided smoking, caffeine, alcohol and strenuous exercise on the study day. Medications were omitted on the morning of the study wherever possible. All vasoactive drugs were withheld for a minimum of 12 h prior to the studies. Participants followed the same protocol for medications at both visits, allowing their results to be compared. All studies were performed in temperature-controlled laboratories (23 ± 1°C), with the participants lying in the supine position following an acclimatisation period of at least 20 min.

### Screening and blood tests

For both baseline and follow-up appointments, screening assessment included anthropometry, electrocardiogram and an interview that included medical history and pharmacotherapy. For anthropometry, height, weight and waist/hip ratio were measured using a standard protocol. Brachial blood pressure was measured in the supine position using an automated blood pressure device (Omron M6; Omron Healthcare Europe, Hoofddorp, the Netherlands) and the mean of three measurements was used for analysis.

All blood tests were measured from a 10 h fasting blood sample collected on the day of the study or within a week of the study day when not possible. Participants’ fasting glucose and lipid concentrations were measured by the hospital’s pathology service (Exeter Pathology Services, Royal Devon and Exeter NHS Foundation Trust), in accordance with the UK National Quality Assessment Scheme. Urinary AER was measured using a timed overnight urine collection protocol and AER was calculated as:

$$ \left[\mathrm{albumin}\ \left(\mathrm{mg}/\mathrm{dl}\right)\times \mathrm{volume}\ \mathrm{of}\ \mathrm{urine}\ \left(\mathrm{dl}\right)\times 1000\right]/\mathrm{time}\ \mathrm{of}\ \mathrm{urine}\ \mathrm{collection}\ \left(\min \right) $$

Albumin was measured by the hospital’s pathology service (Exeter Pathology Services, Royal Devon and Exeter NHS Foundation Trust) in accordance with the UK National Quality Assessment Scheme. Albumin/creatinine ratio was measured from a random spot urine sample taken on the day of one of the visits. Analyses of albumin and creatinine concentrations were performed using immunoturbidimetric and Jaffe methods, respectively, with a detection limit for albumin of 3.0 mg/l. Diabetic retinopathy was graded from two-field photography (nasal and macular view) using the English National Diabetic Retinopathy Grading scheme; for the purposes of this study, diabetic retinopathy was grouped into the following categories: no diabetic retinopathy; nonproliferative diabetic retinopathy; proliferative diabetic retinopathy; and previous laser treatment for proliferative diabetic retinopathy and/or clinically significant macular oedema. Neuropathy was assessed with a vibration perception test on the pulp of the hallux of left and right toes using a neurothesiometer (SLS, Nottingham, UK). A threshold of 25 V was used to define neuropathy.

### Skin microvascular assessments: Iontophoresis of endothelial-dependent (acetylcholine) and -independent (sodium nitroprusside) vasodilators

Response to pharmacological stimulation of the skin blood vessels was conducted as previously described [7]. A solid-state laser Doppler imager (LDI) (LDI2; Moor Instruments, Axminster, UK) was used to measure skin perfusion, with the head of the LDI positioned 50 cm above the skin. The LDI was set to scan a region of 4.8 cm^2^. The LDI was interfaced with a computer equipped with moorLDI software (Research Version 5.3). A battery-powered iontophoresis controller (MIC 1; Moor Instruments) was used to provide the current for iontophoresis.

After very gentle skin cleaning with alcohol wipes, Perspex direct electrode chambers were attached to the volar aspect of the forearm using a double-sided adhesive ring, avoiding visible veins, freckles and hair. Chambers were filled with solutions under investigation; ACh (1% Miochol-E dissolved in mannitol; Novartis, Camberly, UK) and SNP (0.25%) (25 mg/ml Nitropress dissolved in 0.45% saline [77 mmol/l NaCl]; Hospira, Lake Forest, IL, USA). A glass cover slip was placed on the drug chamber to prevent reflection artefacts from light scattered by the convex surface of the solution in the chamber. An indifferent electrode was attached to the volar aspect of the participant’s wrist and connected to the chamber terminal to complete the circuit.

ACh was delivered using five pulses (100 μA each) of anodal current with a 60 s interval between each dose (total charge 12 mC). Perfusion was assessed at rest and then every 20 s for 6 min from the start of the charge period, with no interval between scans.

SNP was delivered by cathodal current. A single pulse of 200 μA was introduced for 60 s (total charge 10 mC). Forearm skin erythrocyte flux was again recorded at rest and then every 20 s for 6 min from the start of the charge period.

Median perfusion response was calculated for each image obtained by the LDI using the total area to which the drug was applied. Skin microvascular perfusion was measured as the area under the response curve, normalised to resting flow, using the trapezius rule for both ACh and SNP and expressed in arbitrary units × min (AU × min).

### Statistical analysis

Analysis was performed on continuous data to maximise power. Normality was formally assessed in variables of interest using the Kolmogorov–Smirnov test against theoretical distribution. Skewed variables were appropriately transformed, and medians [IQRs] are presented. Statistical significance for categorical variables was calculated using the χ^2^ test, and Student’s *t* tests or one-way ANOVA for continuous variables, at baseline and follow-up. Where no appropriate transformation was available, nonparametric alternatives (Mann–Whitney *U* test) were applied and median [IQR] is presented. Differences in change over time between groups were analysed using a two-way ANOVA (time × group) and *p*_interaction_ is reported. Multiple regression was used to investigate the predictors of change over time. In exploring mechanisms for the effect of time, potential confounding effects of age and sex, and mechanistic factors, were considered. Mechanistic factors known to affect microvascular function included blood pressure indices (brachial systolic, brachial diastolic and mean arterial pressure), body size (weight, BMI, waist circumference and waist/hip ratio), insulin resistance (fasting glucose and HbA_1c_) and lipid profile (total cholesterol, LDL-cholesterol, HDL and triacylglycerols). A single model was developed by choosing a variable from each of these groups, based on the greatest increase in the amount of variance explained by their inclusion in the bivariate model against AUC for endothelial-dependent response to ACh. Additional analyses were performed assessing the impact of treatment with agents thought to impact vascular function, including statins, drugs acting on the renin angiotensin system and glucose-lowering drugs. All results of multivariate modelling are presented after adjustment for sex and age at baseline. When considering the principle variables of interest in the microcirculation, a result was deemed significant if *p* ≤ 0.05. When comparing other variables between groups, *p* values should be regarded as indicative.

## Results

Baseline characteristics of the cohort recruited for this study are presented in Table 1. Due to the demographics of the local population, all participants were of white European descent. All of the female participants were postmenopausal at recruitment to the baseline visit. Those with type 2 diabetes were slightly older and had higher BMI and HbA_1c_, but a more favourable lipid profile, than those without diabetes, likely representing more frequent statin prescription. Blood pressure was similar in those with and without diabetes, although almost twice as many with diabetes were receiving antihypertensive therapy. People with type 2 diabetes received a variety of glucose-lowering treatments: 20.1% were treated with diet only, 61.7% received oral glucose-lowering medication, 5.2% received insulin and 13.0% were treated with a combination of oral medication and insulin.

**Table 1:** Baseline characteristics of the cohort stratified by the presence of diabetes (DM) or absence of diabetes (No DM)

Characteristic	No DM (*n* = 99)	DM (*n* = 154)	*p*
Sex (male/female) (*n*)	41/58	42/112	0.019
Age (years)	64.8 (63.0, 66.5)	67.9 (66.6, 69.2)	0.004
Weight (kg)	75.6 (72.9, 78.3)	91.2 (88.6, 93.8)	<0.001
Height (m)	1.70 (1.68, 1.72)	1.72 (1.71, 1.73)	0.145
BMI (kg/m^2^)^a^	25.7 [23.8–27.8]	30.2 [27.6–33.6]	<0.001
Waist circumference (cm)	93.1 (90.9, 95.3)	106.9 (104.9, 108.9)	<0.001
Systolic BP (mmHg)	137 (133, 141)	138 (136, 140)	0.664
Diastolic BP (mmHg)	75.6 (73.8, 77.4)	76.2 (74.8, 77.5)	0.654
MAP (mmHg)	96.1 (93.9, 98.3)	96.7 (95.3, 98.2)	0.619
ABPI right	1.15 (1.12, 1.18)	1.15 (1.12, 1.17)	0.83
ABPI left	1.14 (1.11, 1.16)	1.13 (1.11, 1.16)	0.8
Total cholesterol (mmol/l)^a^	4.80 [4.10–5.70]	3.90 [3.30–4.50]	<0.001
LDL-cholesterol (mmol/l)^a^	2.70 [1.98–3.31]	1.88 [1.53–2.38]	<0.001
HDL-cholesterol (mmol/l)^a^	1.53 [1.23–1.92]	1.24 [1.06–1.43]	<0.001
HbA_1c_ (mmol/mol)^a^	40 [38–42]	57 [49–67]	<0.001
HbA_1c_ (%)^a^	5.7 [5.5–6.0]	7.4 [6.6–8.3]	<0.001
History of CVD (%)	43.4	47.4	0.536
On antihypertensive (%)	40.4	79.2	<0.001
β-blocker	21.4	33.8	0.035
ACE-inhibitor	20.4	51.3	<0.001
Angiotensin receptor A	6.1	17.5	0.009
On statin therapy (%)	45.9	81.8	<0.001
Smoking status (%)			
Current	5.1	4.6	
Previous	48.0	50.7	
Never	46.9	44.8	0.911
AER (μg/min)^a^	4.49 [3.01–6.07]	5.71 [3.88–11.65]	<0.001
ACR (mg/mmol)^a^	0.72 [0.53–1.10]	1.07 [0.59–2.17]	0.014
Retinopathy (*n*)			
No retinopathy	NA	87	NA
Nonproliferative retinopathy	NA	52	NA
Proliferative	NA	0	NA
Laser treatment	NA	4	NA
Unknown	NA	11^b^	NA
Neuropathy (%)	NA	24^c^	NA

Data are displayed as mean (95% CI) or median IQR

^a^Skewed variable; median and IQR presented and *p* for difference of the appropriately transformed data

^b^Unknown retinopathy score owing to ungradable two-field photography or data not available on clinical database

^c^Defined as neurothesiometer measurement ≥25 V, available in 146 of 154 participants with type 2 diabetes

ABPI, ankle brachial pressure index; ACR, albumin/creatinine ratio; F, female; M, male; MAP, mean arterial pressure; NA, not applicable

Data for change in metabolic and microvascular variables over 3 years are presented in Table 2. AER was higher at baseline and follow-up in those with diabetes compared with those without; however, this was below the range for clinically significant microalbuminuria (Table 2; difference between those with and those without type 2 diabetes at both baseline and follow-up, *p* < 0.001). Over 3 years, there was no significant increase in AER at this preclinical level, nor was there any difference in change over 3 years between those with and those without diabetes (*p*_interaction_ = 0.330).

**Table 2:** Change in metabolic and microvascular variables over 3 years stratified by the presence of diabetes

Variable	No diabetes (*n* = 99)				Type 2 diabetes (*n* = 154)				
	Baseline	Follow-up	Δ	*p*	Baseline	Follow-up	Δ	*p*	*p*_interaction_
Age (years)	64.8 (63.0, 66.5)	67.9 (66.2, 69.6)	3.1	0.012	67.9 (66.6, 69.2)*	71.0 (69.7, 73.3)*	3.1	<0.001	0.990
Weight (kg)	75.6 (72.9, 78.3)	75.4 (72.6, 78.1)	−0.2	0.940	91.2 (88.6, 93.8)***	89.5 (87.0, 92.1)***	−1.7	0.370	0.566
BMI (kg/m^2^)	25.7 [23.8–27.8]	25.6 (25.1, 26.8)	0.1	0.553	30.2 [27.6–33.6]***	30.4 (29.7, 31.2)***	0.0	0.803	0.799
Waist circumference (cm)	93.1 (90.9, 95.3)	91.6 (89.5, 94.4)	−1.5	0.508	106.9 (104.9, 108.9)***	106.8 (104.8, 108.8)***	−0.1	0.939	0.611
Systolic BP (mmHg)	137 (133, 141)	132 (129, 136)	−5.0	0.069	138 (136, 140)	135 (129, 136)	−3.0	0.114	0.496
Diastolic BP (mmHg)	75.6 (73.8, 77.4)	70.4 (68.8, 72.0)	−5.2	<0.001	76.2 (74.8, 77.5)	70.4 (69.1, 71.6)	−5.8	<0.001	0.679
MAP (mmHg)	96.1 (93.9, 98.3)	91.1 (89.2, 93.0)	−5.0	<0.001	96.7 (95.3, 98.2)	92.0 (90.6, 93.3)	−4.7	<0.001	0.900
Total cholesterol (mmol/l)^a^	4.80 [4.10–5.70]	4.90 [4.00–5.85]	0.10	0.711	3.90 [3.30–4.50]***	3.90 [3.40–4.5]***	0.0	0.888	0.389
LDL-cholesterol (mmol/l)^a^	2.70 [1.98–3.31]	2.69 [2.00–3.42]	−0.01	0.827	1.88 [1.53–2.38]***	1.93 [1.57–2.44]***	0.05	0.742	0.954
HDL-cholesterol (mmol/l)^a^	1.53 [1.23–1.92]	1.61 [1.29–1.95]	0.08	0.281	1.24 [1.06–1.43]***	1.26 [1.05–1.46]***	0.02	0.545	0.193
HbA_1c_ (mmol/mol)^a^	40 [38–42]	40 [37.5–43.0]	0.0	0.398	57 [49–67]***	56.5 [49–66]***	−0.5	0.695	0.114
HbA_1c_ (%)^a^	5.7 [5.5–6.0]	5.7 [5.4–6.1]	0.0	0.398	7.4 [6.6–8.3]***	7.4 [6.6–8.3]***	0.0	0.695	0.114
ACR (mg/mmol)^a^	0.59 [0.35–0.91]	0.66 [0.43–1.21]	0.07	0.036	0.69 [0.47–1.4]*	0.97 [0.488–3.0]*	0.28	0.088	0.005
AER (μg/min)^a^	4.49 [3.01–6.07]	4.21 [2.82–6.04]	0.28	0.596	5.71 [3.88–11.65]***	6.06 [4.1–12.39]***	0.35	0.482	0.330
Response to ACh (AU × min)	111.9 (102.3, 121.4)	95.4 (87.5, 103.3)	16.5	0.009	93.9 (88.1, 99.4)***	79.7 (73.0, 86.4)*	14.2	0.002	0.740
Response to SNP (AU × min)	75.1 (67.8, 82.4)	62.4 (56.0, 68.8)	12.7	0.01	63.2 (59.2, 67.2)*	48.3 (43.7, 52.9)***	14.9	<0.001	0.691

Data are displayed as mean (95% CI) or median [IQR]

^a^Skewed variable; median and IQR presented and *p* for difference in change of variables over 3 years, and for difference between change in those with and without diabetes calculated using Mann–Whitney *U* test

*p < 0.05 vs those without diabetes at the same time point

***p < 0.001 vs those without diabetes at the same time point

ABPI, ankle brachial pressure index; ACR, albumin/creatinine ratio; MAP, mean arterial pressure; Δ, difference between follow-up and baseline measurements

**Fig. 1:**
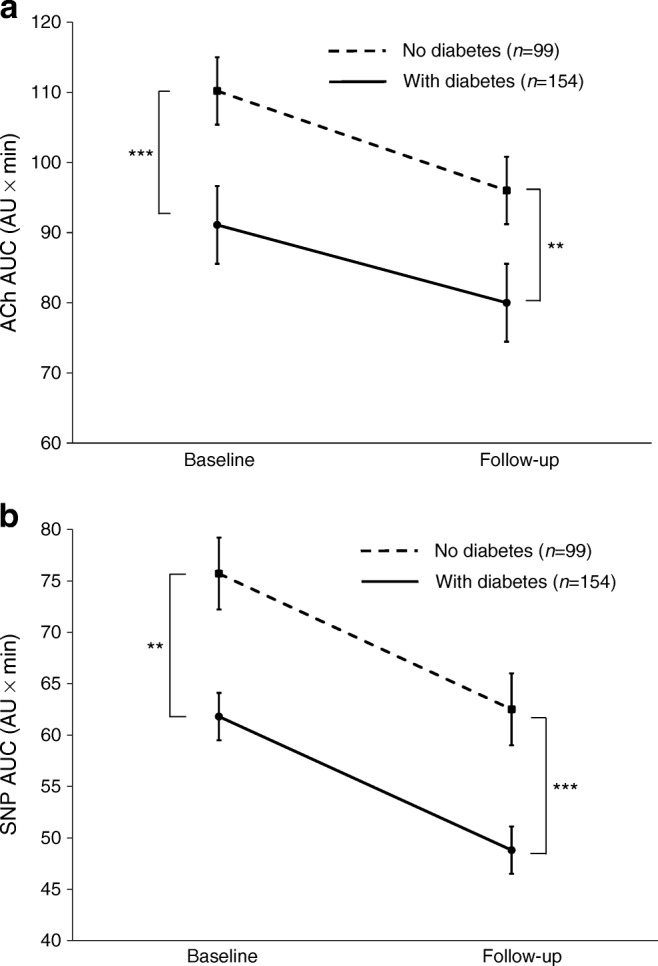
Endothelial-dependent (**a**) and -independent (**b**) microvascular response at baseline and follow-up in participants with and without diabetes, over a 3 year period, measured as microvascular perfusion in response to Ach (**a**) and SNP (**b**). Data are presented as the mean AUC of the response curve (AU × min ± SEM). Two-way ANOVA (grouped by time) was used to test interaction (**a***p* = 0.740; **b***p* = 0.691). (**a**) People with diabetes had attenuated microvascular function in response to ACh at baseline (***p < 0.001) and follow-up (**p < 0.01). There was significant change in ACh over 3 years in people both with (*p* = 0.002) and without type 2 diabetes (*p* = 0.009). (**b**) People with diabetes had attenuated microvascular function in response to SNP at baseline (**p < 0.01) and follow-up (***p < 0.001). There was significant reduction in SNP over 3 years in people both with (*p* < 0.001) and without type 2 diabetes (*p* = 0.01)

At both baseline and follow-up, endothelium-dependent (ACh) and -independent (SNP) microvascular responses were attenuated in people with type 2 diabetes compared with those without (Table 2, Fig. 1a,b). Over 3 years, there was a similar decline in mean microvascular function in the groups with and without diabetes.

### Determinants of change in microvascular function over the 3 years

Although the average decline in group mean microvascular function was similar in individuals both with and without diabetes (age- and sex-adjusted standardised β = 0.016, *p* = 0.8; and −0.05, *p* = 0.4 for change in ACh and SNP, respectively), there was significant diversity of magnitude and direction of change within the populations. Exploring the whole cohort, percentage change in weight accounted for the greatest variance of the model, making it the single most predictive factor (Table 3) for the decline in microvascular function, such that a reduction in weight was associated with less of a deterioration of both endothelium-dependent and -independent microvascular function, whereas weight gain was associated with a greater decline in function. All parameters of change in body composition and adiposity, including absolute change in weight, change in BMI and change in waist/hip ratio, gave similar results. Those on statin therapy saw less of a decline in endothelium-dependent function compared with those not on statin therapy. There was no association between change in microvascular function and history of CVD, change in any blood pressure variable, any blood pressure intervention (including β-blockers or modifiers of the renin angiotensin aldosterone system), change in HDL-cholesterol or change in LDL-cholesterol.

**Table 3:** Association of changes in microvascular function with conventional cardiovascular risk factors and treatment in the whole population and in those with and without diabetes after adjustment for age and sex

Variable	Change in ACh		Change in SNP	
	Standardised β	*p* value	Standardised β	p value
Whole cohort				
History of CVD	0.069	0.292	−0.121	0.065
Percentage change in weight	−0.248	<0.001	−0.165	0.009
Change in diastolic blood pressure	−0.058	0.374	−0.030	0.647
Change in LDL-cholesterol	0.103	0.138	0.071	0.306
Statin treatment at baseline	0.160	0.016	−0.015	0.821
Diabetes-specific analysis (*n* = 154)^a^				
History of CVD	0.020	0.808	−0.133	0.112
Percentage change weight	−0.317	<0.001	−0.240	0.003
Change in diastolic blood pressure	−0.076	0.363	−0.056	0.504
Change in LDL-cholesterol	0.016	0.853	0.034	0.704
Percentage change in HbA_1c_	−0.203	0.014	0.082	0.329
Sulfonylurea treatment^b^	−0.199	0.022	−0.112	0.209
People without diabetes (*n* = 99)				
History of CVD	0.183	0.084	−0.095	0.370
Percentage change in weight	−0.160	0.117	−0.031	0.764
Change in diastolic blood pressure	−0.037	0.726	−0.002	0.987
Change in LDL-cholesterol	0.235	0.033	0.109	0.313
Statin treatment at baseline	0.190	0.080	−0.137	0.209

Standardised β (i.e. SD change in variable of interest per SD change in contributing factor) from multivariate modelling between the microcirculatory variable of interest and the mechanistic/confounding factor after adjustment for age and sex. A negative standardised β represents a greater decline in microvascular function per increase in variable (e.g. per SD increase in weight there will be ~1/4 of an SD greater decline in endothelial-dependent microvascular function over 3 years)

^a^Statin therapy not considered for people living with diabetes due to co-linearity with 81% of individuals being treated

^b^In a model with all glucose-lowering drugs available accounted for

In those with diabetes alone, change in weight was the strongest predictor of changes in endothelial-dependent and -independent microvascular function (Table 3). Numerically, this was greater by approximately a quarter for endothelial-dependent and 45% for endothelial-independent function than in those without diabetes, although this interaction was not statistically significant (*p*_interaction_ = 0.6 and 0.5 for ACh and SNP, respectively). Change in glycaemic control (HbA_1c_) was also associated with a modest change in endothelial-dependent microvascular function over time, such that an increase in HbA_1c_ was associated with a poorer function independent of change in weight. Within a small cohort of participants, interim HbA_1c_ values were available. For these individuals, we stratified into those with ‘good control’, with a mean HbA_1c_ < 53 mmol/mol (7.0%) over the 3 years, and those with less good control, with a mean HbA_1c_ > 64 mmol/mol (8.0%; electronic supplementary material [ESM] Fig. 1). Those with good glycaemic control had less attenuation of microvascular function over the 3 years compared with those with poorer control (*p* = 0.03); however, this was accounted for by less weight gain in those with better glycaemic control (*p* after adjustment 0.2). The use of sulfonylureas (*n* = 46, 29.9% of the population with diabetes) was associated with a greater decline in endothelial microvascular function compared with those with diabetes on other therapies, including insulin, after adjustment for the HbA_1c_ effect (adjusted standardised β 0.199, *p* = 0.02). This association was independent of change in weight, other diabetes therapies and duration of diabetes (adjusted standardised β 0.198, *p* = 0.02). An exploratory analysis determining the impact of going into remission from diabetes (defined as HbA_1c_ < 48 mmol/mol [6.5%] on no glucose-lowering medications) suggested a numerical improvement in endothelial function compared with those that remained with diabetes (ESM Table 1). However, no formal statistical analysis was performed for this exploratory analysis due the small numbers.

### Thresholds of benefit

To further explore the association between weight change and change in microvascular function, we divided our cohort into participants who, over the 3 years of the study, had increased, decreased or stable weight, defined as 5% or less variation (loss or gain) in their weight. The 5% threshold in weight reduction has recently been shown to reduce risk of cardiovascular events in patients suffering with type 2 diabetes [21]. One hundred and ninety participants had a stable weight and 40 participants lost 5% or more of their weight (median 8% weight loss), including one participant who underwent bariatric surgery, whereas 22 participants gained 5% or more of their weight (median 7.3% weight gain).

**Fig. 2:**
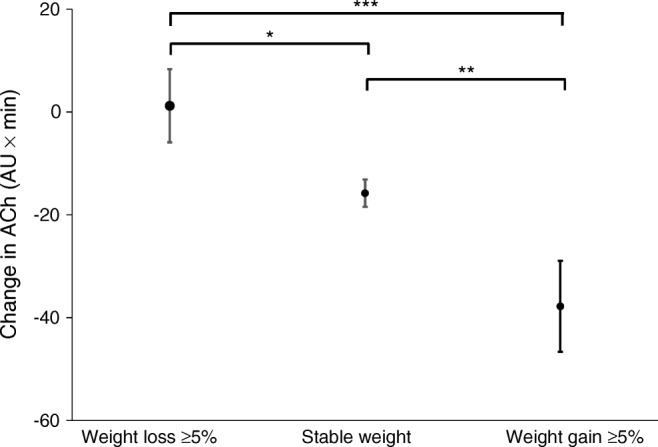
Change in AUC in response to endothelial-dependent (ACh) stimulation over 3 years stratified into those that had ≥5% weight loss, those that were weight stable and those that gained ≥5% of their weight (AU × min ± SEM). *p* values from *t* tests, *p < 0.05, **p < 0.01 and ***p < 0.001

Endothelial-dependent microvascular response to ACh did not decline in those who lost ≥5% over the 3 years compared with those who had stable weight, whereas those who gained ≥5% weight had an exaggerated attenuation in microvascular function (change in endothelial-dependent function was 1.2 [95% CI −13.2, 15.7] AU × min in those who lost weight; −15.8 [−10.5, −21.0] AU × min in those with stable weight; and −37.8 [−19.4, −56.2] AU × min in those who put on ≥5% weight; Fig. 2). Adjustment for age, sex, changes in blood pressure, blood glucose and cholesterol did not change the association (ESM Table 2). These differences were numerically similar in those with and without diabetes, although, possibly due to the reduced power in the smaller number without diabetes, the significance was only maintained in those with diabetes (*p* = 0.025 for weight loss vs stable weight, *p* < 0.001 for weight loss vs weight gain and *p* = 0.007 for weight gain vs weight stable). The effect of weight loss in those with diabetes resulted in an endothelial-dependent microvascular response after 3 years that was similar to those without diabetes who had stable weight over this time period (response in people with diabetes who lost weight = 101.8 AU × min vs people without diabetes who maintained their weight 94.1 AU × min). No formal testing was done on these comparisons.

For endothelial-independent response to SNP in the whole group, there was only a trend towards difference between those who lost ≥5% over the 3 years and those who had stable weight (*p* = 0.1). This trend appeared to be driven entirely by a significant difference in those with diabetes, such that those who lost ≥5% weight had a lower decline in SNP response compared with those who remained weight stable (−4.5 [4.6, −13.6] vs −16.6 [−12.0, −21.2] AU × min; *p* = 0.02) and those who gained weight (−4.5 [4.6, −13.6] vs −21.3 [−9.2, −33.4] AU × min; *p* = 0.03). There was no difference between those who had stable weight and those who gained weight (*p* = 0.43).

## Discussion

We have demonstrated for the first time that, over a 3 year period, endothelial-dependent microvascular function declines in weight-stable individuals, but that this decline is attenuated with a modest amount of weight loss and accelerated with a modest amount of weight gain. This was found in a mixed cohort of people with and without type 2 diabetes. Importantly, our findings were independent of the measures of blood pressure and cholesterol we used and also independent of the treatment regimens. In individuals with type 2 diabetes, change in weight and change in HbA_1c_ were independent predictors of change in endothelial-dependent response, but only change in weight was associated with change in endothelial-independent response.

The attenuation of decline in ACh responses in the absence of an impact on SNP response in this study suggests that weight loss is associated with an improvement in endothelial function. This is in keeping with the growing understanding that obesity impairs endothelial function through systemic vascular inflammation [22].

Increased BMI and adiposity have an adverse effect on the microcirculation, independent of diabetes, in part due to the cellular inflammatory response to ischaemia occurring once the hypertrophy of adipocytes is beyond the 150 μm diffusion range of oxygen [23]. Previous studies have shown that weight loss is associated with improvements in cyclical changes in microvascular perfusion (vasomotion) [24] and decreased proteinuria [25], but these studies involved significant weight loss interventions such as gastric banding and bypass surgery. Unlike these previous investigations, the 5% weight loss that demonstrated benefit within our study is of modest magnitude and achievable without drastic interventions. In fact, we have shown that a weight reduction of at least 5% or more of body weight was associated with clear benefits in endothelial-dependent microvascular function.

A weight loss of at least 5% has been shown to have other beneficial effects in type 2 diabetes. Recently, for example, this weight change threshold has been shown to be associated with improvement in HbA_1c_ (a finding replicated here) and a lower 10 year hazard of CVD [21] in newly diagnosed type 2 diabetes patients. Our previous results [15] support the results of Strelitz et al [21], suggesting that the benefits of a 5% weight loss are wide ranging and that weight loss should be strongly encouraged.

This study has also shown that, in people living with diabetes, improving glycaemic control is associated with a lower decline in microvascular function over 3 years. It is important to consider whether this represents a mechanistic pathway, or simply confounding based on shared risk factors. However, this finding is supported by previous studies which demonstrated that tight glycaemic control reduced microvascular complications [26, 27]. Our study significantly adds to the current knowledge by demonstrating that these benefits can be realised at a stage prior to patients having microvascular complications, and therefore should be encouraged as soon as possible for people living with diabetes.

We found no difference in change in microvascular function over 3 years between people with and without diabetes. This was an unexpected finding; however, the association between direction of glycaemic control and microvascular function suggests that the absence of a difference may be due to population-wide improvement in glycaemic control in the group of individuals with diabetes, and a move away from sulfonylurea therapy over the 3 years. It is also important to note that, in the individuals with diabetes, lower microvascular function at baseline remained poorer at follow-up. The benefit of weight loss appeared to be exaggerated in those with diabetes compared with those without, although this difference did not reach statistical significance. There are several potential explanations for this. It may be that the lower baseline microvascular function in those with diabetes gives more potential for benefit of weight loss. There may be additional benefit in people with diabetes, given that insulin resistance is dependent on obesity, and thus weight loss improves the microvascular function directly and indirectly through improvement in the nonglycaemic elements of diabetes. Finally, it may simply be coincidental, given that the study was not powered to explore differences between those with and without diabetes but rather to assess the impact of diabetes itself.

### Strengths and limitations

To our knowledge, this is the largest study to have followed the natural history of microvascular function in people with and without diabetes over 3 years, who are treated in accordance with common practice within the UK. By evaluating a general population sample, with and without CVD, unlike the tight control that is delivered in randomised controlled trials, these results are representative of what a healthcare provider can reasonably expect to achieve working with their patient population. The observational nature, however, means it is impossible to ascribe causation. There also remains the potential for systematic bias, in terms of health-seeking behaviour and adherence to therapies prescribed. This is more likely to be greater in populations that also adhere to lifestyle suggestions. As a result, weight loss may simply be a proxy for medication adherence. Additionally, the study cannot exclude shared confounding of weight loss and improvement in microvascular endothelial function from other, unmeasured mechanistic factors, although the failure of conventional risk factors to account for these associations, having only a minimal effect on the standardised βs, makes this unlikely. Further, as a secondary analysis of a subpopulation from the SUMMIT cohort, study power was limited for subgroup analyses. Across the whole population with the complete microvascular dataset, we have 97% power to detect a clinically meaningful 7% variance (*R*^2^) that was accounted for by difference in weight. Within the diabetes subpopulation, however, this power is reduced to 84%, and further reduced to 64% for those without diabetes. This likely accounts for the lack of significant benefit demonstrated in the population without diabetes, despite similar numerical benefit as in those with diabetes.

### Conclusion

In conclusion, we have demonstrated that modest weight loss and good glycaemic control are associated with less 3 year decline in microvascular function in people with diabetes. Unlike studies in laboratory settings, where experimental conditions are tightly controlled, we observed the microvascular progression in a large group of people managed in the community by their usual physicians and no additional study-specific interventions. Further, we have demonstrated that a modest weight loss of only 5% attenuated the time-dependent decline of endothelial-dependent microvascular function over 3 years.

## Electronic supplementary material

ESM (PDF 158 kb)

## Data Availability

Original data are available by direct application to the corresponding author.

## Abbreviations

ACh: Acetylcholine
AU × min: Arbitrary units × min (units for AUC of the response curve)
CVD: Cardiovascular disease
LDI: Laser Doppler imager
NHS: National Health Service
SNP: Sodium nitroprusside
SUMMIT: SUrrogate markers for Micro- and Macro-vascular hard endpoints for Innovative diabetes Tools
