# Supplementary material for: Weight change and sulfonylurea therapy are related to 3 year change in microvascular function in people with type 2 diabetes
Source: Diabetologia. 2020 Mar 17;63(6):1268–78. doi: 10.1007/s00125-020-05125-4 (PMC7228909; doi:10.1007/s00125-020-05125-4)
Supplement: Supplementary file 1 — (PDF 158 kb) [file 125_2020_5125_MOESM1_ESM.pdf]

ESM Table 1 The impact of new diagnosis diabetes or going into remission (defined as a HbA1c of <48mmol/mol whilst on no antihyperglycaemic medications) on the microvascular responses to ACh and SNP (median and IQ range) No formal statistical analysis performed due to low numbers in this exploratory analysis.

	Change in ACH (AU)	Change in SNP (AU)
Remission (*n*=10)	3.8 [-25.2, 18.2]	-16.5 [-31.4, 2.7]
Type 2 diabetes (*n*=144)	-17.8 [-34.3, 6.7]	-16.5 [-30.6, 0.2]
Newly diagnosed (*n*=8)	-14.2 [-40.2, -4.9]	-7.1 [-22.4, 0.3]

Supplementary Table 2.
Attenuation in endothelial dependent (ACh mediated) microvascular function (mean(SEM)) after 3 years, stratified by weight loss, weight neutrality and weight gain after adjustment for potential confounding and mechanistic factors

**Adjusted for**	≥5% weight loss (n=40)	Weight neutral (n=191)	≥ 5% weight gain (n=22)	p for trend
Age & Sex	+1.5 (6.1)	-16.0 (2.1)	-36.3 (8.3)	<0.0001
As above & change in diastolic blood pressure	+1.2 (6.2)	-15.9 (2.8)	-36.3 (8.6)	<0.001
As above and change in LDL cholesterol	+5.6 (6.7)	-15.6 (3.0)	-33.3 (9.0)	<0.001
As above and Statin treatment	+6.0 (6.6)	-15.6 (2.9)	-34.3 (8.7)	<0.001
As above and presence of diabetes	+6.3 (6.6)	-15.7 (2.9)	-33.8 (8.8)	<0.001
As above and history of CV disease	+6.5 (6.7)	-15.7 (2.9)	-34.1 (8.9)	<0.001
**Subgroup with diabetes**	**N=28**	**N=110**	**N=16**	
Age & Sex adjusted	+1.2(6.4)	-14.2(3.3)	39.4 (8.7)	<0.001
**Subgroup without diabetes**	**N=12**	**N=81**	**N=6**	
Age & Sex adjusted	+1.5 (13.2)	-18.0 (5.1)	-32.3 (18.7)	0.1

Supplementary Figure 1. Change in total endothelial dependent (figure 2a) and endothelial independent (figure 2b) microvascular response over 3 years in those with diabetes who had a minimum of annual HbA1c results available, stratified into those that had a mean HbA1c ≤53mmol/mol (7.0%) compared to those with a mean HbA1c >64mmol/mol (8.0%)

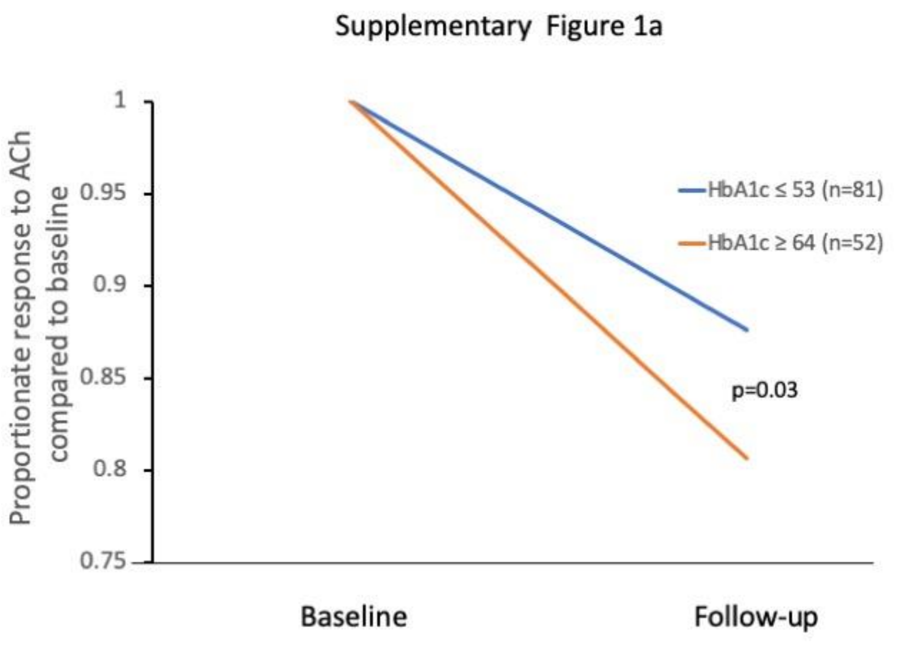

Supp Figure 1a. Exploratory analysis of association between HbA1c control over the 3-year period and changes in microvascular endothelial dependent response to Acetyl Choline (ACh), in the sub population that annual HbA1c was available for.

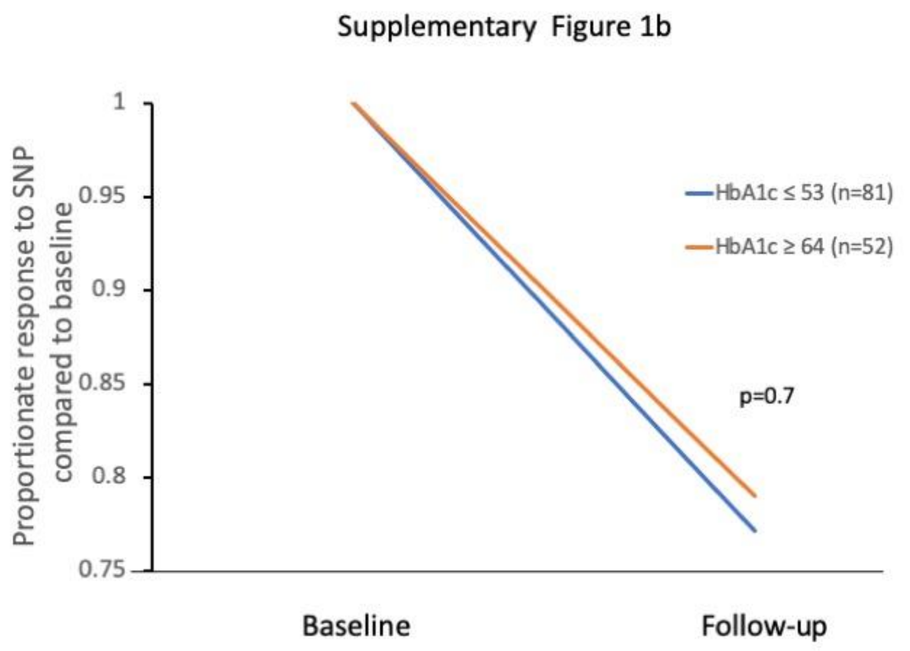

Supp Figure 1b. Exploratory analysis of association between HbA1c control over the 3-year period and changes in microvascular endothelial independent response to Sodium Nitroprusside (SNP), in the sub population that annual HbA1c was available for.
